# Perceived Stress, Sources and Severity of Stress among medical undergraduates in a Pakistani Medical School

**DOI:** 10.1186/1472-6920-10-2

**Published:** 2010-01-15

**Authors:** Mohsin Shah, Shahid Hasan, Samina Malik, Chandrashekhar T Sreeramareddy

**Affiliations:** 1 3rd year Undergraduate Medical Student, CMH Lahore Medical College, University of Health Sciences, Abdur Rahman Road, Lahore Cantt, Pakistan; 2 Department of Physiology, CMH Lahore Medical College, University of Health Sciences, Abdur Rahman Road, Lahore Cantt, Pakistan; 3 Department of Community Medicine, Melaka Manipal Medical College, Malaysia

## Abstract

**Background:**

Recently there is a growing concern about stress during undergraduate medical training. However, studies about the same are lacking from Pakistani medical schools. The objectives of our study were to assess perceived stress, sources of stress and their severity and to assess the determinants of stressed cases.

**Methods:**

A cross-sectional, questionnaire-based survey was carried out among undergraduate medical students of CMH Lahore Medical College, Pakistan during January to March 2009. Perceived stress was assessed using the perceived stress scale. A 33-item questionnaire was used to assess sources of stress and their severity.

**Results:**

The overall response rate was 80.5% (161 out of 200 students). The overall mean perceived stress was 30.84 (SD = 7.01) and was significantly higher among female students. By logistic regression analysis, stressed cases were associated with occurrence of psychosocial (OR 5.01, 95% CI 2.44-10.29) and academic related stressors (OR 3.17 95% CI 1.52-6.68). The most common sources of stress were related to academic and psychosocial concerns. 'High parental expectations', 'frequency of examinations', 'vastness of academic curriculum', 'sleeping difficulties', 'worrying about the future', 'loneliness', 'becoming a doctor', 'performance in periodic examinations' were the most frequently and severely occurring sources of stress. There was a negative but insignificant correlation between perceived stress and academic performance (r = **-**0.099, p > 0.05).

**Conclusion:**

A higher level of perceived stress was reported by the students. The main stressors were related to academic and psychosocial domains. Further studies are required to test the association between stressed cases and gender, academic stressors and psychosocial stressors.

## Background

Recently stress during medical training is increasingly being reported in published literature. Previous studies have shown fairly high levels of distress, such as symptoms of depression [1,2] and even suicide thoughts [3,4] among medical undergraduates. The potential negative effects of emotional distress on medical students include impairment of functioning in class-room performance and clinical practice, stress-induced disorders and deteriorating performance [5,6]. Perceived medical stress has also been linked to current mental distress [7] and to forthcoming health problems [8]. However, there is very little information about the effect of stress on academic performance during medical training [9].

In addition to stress the students' social, emotional and physical as well as family problems may influence their learning ability and academic performance [10,11]. Higher levels of stress may have a negative impact on the students learning ability. Excessive stress may result in mental and physical problems and may diminish a student's sense of worth and might affect his/her academic achievement [12,13]. Earlier studies have classified stressors into three main categories: academic pressures, social issues and financial problems [14]. Studies from developing countries like Thailand, India, Malaysia and even Pakistan have reported stress among medical students and have also underscored the role of academics as a potential stressor [15-18]. But these studies either did not use the perceived stress scale or did not study the correlation between stress and academic performance [19]. Studies have reported that academic constraints, [20] and also factors like age, gender, ethnicity and marital status may also influence students' severity of stress and hence academic performance [21,22].

At the Combined Military Hospital Medical College (CMH LMC), Lahore, Pakistan, there are students from Pakistan, Saudi Arabia, United States, U.A.E and other countries. These students come from diverse cultural, socioeconomic and educational backgrounds. These foreign nationals who account for more than a third of the student population are exposed to a new learning environment, making new social circles and also adapting to a new and different world during their training at CMH LMC. This may be a very stressful experience especially during the formative first and second years of their course. The relative paucity of information about stress and its sources during the early years of medical undergraduate training in Pakistan warranted this study. The objectives of our study were:

1. To assess perceived stress, sources of stress and their severity.

2. To assess the determinants of stressed cases.

## Methods

### Setting and participants

The present study was undertaken at CMH LMC, affiliated to University of Health Sciences (UHS), Lahore. The three basic science subjects (Anatomy, Physiology and Biochemistry) are taught during first two years of MBBS (Bachelor of Medicine and Bachelor of Surgery) course in a lecture-based region wise manner. Each year CMH LMC admits a batch of 100 students for the MBBS course. At the end of each academic year the students sit for their professional examinations, held by the UHS, to qualify for the next academic year. The students of first and the second year MBBS admitted during 2006 and 2007 respectively were invited to participate in this study

### Study Design

A cross-sectional survey using self-administered questionnaire. The institutional ethics committee of CMH LMC approved this study. A total of 200 medical undergraduates from the first and second year MBBS batches at CMH Lahore Medical College were included for survey. We did not have any earlier estimates of prevalence of stress among medical students in Pakistan. Therefore we assumed the prevalence to be 50% and calculated the required sample for our survey with an allowable error of 15%, 95% confidence limits and 10% for non-responders. Thus the final sample size required was 187. The students were asked to complete a set of questionnaires consisting of three parts namely: demographic information and academics, (Appendix 3) perceived stress scale [19] (Appendix 4), a 33-item list of potential stressors (Appendix 5).

## Definitions of variables

### Perceived stress

Perceived stress was measured using the perceived stress scale (PSS-14) [19], which comprised of 14 questions with responses varying from 0 to 4 for each item and ranging from never, almost never, sometimes, fairly often and very often respectively on the basis of their occurrence during one month prior to the survey. The PSS has an internal consistency of 0.85 (Cronbach α co-efficient) and test-retest reliability during a short retest interval (several days) of 0.85 [19]. It assesses the degree to which participants evaluate their lives as being stressful during the past month. It does not tie appraisal to a particular situation; the scale is sensitive to the nonoccurrence of events as well as ongoing life circumstances. PSS-14 scores are obtained by reversing the scores on four positive items, for example 0 = 4, 1 = 3, 2 = 2, etc. and then summing across all 14 items. Items 4, 5, 6, 7 and 10 are the positively stated items. The scale yielded a single score with high scores indicating higher levels of stress and lower levels indicating lower levels of stress. The PSS-14 has a possible range of scores from 0 to 56. The range of PSS scores were also divided into stratified quartiles. The upper two and lower two quartiles were combined (28 being the operational cut off value for the upper bound) and were labeled as stressed and not stressed respectively. This cut off value was selected in accordance to a similar study from Egypt [23].

### Academic performance

Academic performance was measured using the student's self reported UHS professional examination score. The total university examination score (marks) was out of 550. The examination score was measured using an open ended question and the possible range of scores could have been from 0 to 550.

### Stressors

The potential stressors in the questionnaire were adapted from a similar study from Nepal by Sreeramareddy et al [24]. A total of 33 stressors were listed and were grouped as academic, psychosocial and health related. For each potential stressor the frequency of occurrence was classified as never, rarely, sometimes, often and always and scored as 1,2,3,4 and 5 respectively. The severity of each stressor was rated using a Likert scale (1-10) ranging from not severe to very severe. The students were asked to indicate if any of the stressors had been affecting them.

### Data collection

One hundred students each from first and second year MBBS admitted during 2006 and 2007 respectively were invited to participate in this survey. The students were given a copy of the written instructions and objectives of the study (Appendix 1). Informed consent was taken from all the participants (Appendix 2). The participants were assured of confidentiality of the information provided and had an option of refusal to participate in the survey. The anonymous questionnaire was distributed amongst students during breaks from their teaching schedule and the researchers collected the completed questionnaires.

### Data analysis

The data was analysed using Statistical Package for Social Sciences (SPSS) 17.0 for Windows (SPSS, Inc., Chicago, IL, USA). The mean score of perceived stress were calculated. The number and percentage of stressed cases were calculated according to demographic variables. We grouped the frequency of occurrence of stressors as never/rarely, sometimes, and often/always. Percentage frequency of occurrence was calculated for each of the stressors from academic, psychosocial and health domains. Descriptive statistics were calculated for severity of stressors. Pearson product-momentum correlation was applied to test the correlation between perceived stress (PSS score) and self-reported academic performance. Logistic regression analyses were carried out to assess determinants of stressed cases. We considered perceived stress (stressed cases) as the dependent variable, demographic variables and groups of stressors (i.e. academic, psychosocial and health-related) as the independent variables. Adjusted odds ratios (OR), 95% confidence intervals (95% CI) were calculated. A p-value < 0.05 was considered as significant.

## Results

### Demographic characteristics of the respondents

Out of 200 students 161 completed and returned the questionnaire giving an overall response rate of 80.5% (78% for first year and 83% for second year). The mean age was 20.35(SD = 1.09) with a range of 17-27 years. Fifty three students were male (32.92%) and 108 were females (67.08%). Among the nationalities 121(75.16%) were Pakistani and 40 (24.8%) were foreign nationals [Saudi Arabia (13), USA (11), U.A.E (6), Bahrain (3), Oman (4), Canada (1), Kuwait (1) and Qatar (1)]. Among the Higher Secondary School Certification 114 had completed their Fellow of science (Fsc) (69.57%), 40 had done A-levels (24.84%) while nine had completed their High School Diploma (5.59%). 148 students had gone to English medium schools (91.93%) whereas only 13 had attended Urdu (a local vernacular language) medium school (8.07%). Ninety nine students were day scholars (61.49%) while 62 (38.51%) were residing in the hostels (Boarders). One hundred and thirty two (81.99%) students reported as being single (81.99%), 29 (18.01%) were involved in active relationships while none were married.

### Perceived stress

Mean PSS score in the study population was 30.84(SD = 7.01) with a median of 31.00 (IQR 26-36). Mean PSS score for female students (n = 108) was 31.94 (SD 6.28) while the same for male students (n = 53) was 28.60 (SD 7.92). The 14-question survey instrument's sample response frequencies are given (Table 1). Using univariate analysis, only gender was significant (*p *< 0.05) with PSS score; the female students reported significantly higher levels of perceived stress than their male counterparts.

**Table 1:** Medicine students' responses to the perceived stress scale

Statement	Never	Almost never	Sometimes	Often	Very often
In the last month, how often have you been upset because of something that happened unexpectedly?	8 (5)	19 (12)	60 (37)	40 (25)	34 (21)
In the last month, how often have you felt that you were unable to control the important things in your life?	17 (11)	26 (16)	55 (34)	36 (22)	27 (17)
In the last month, how often have you felt nervous and "stressed"?	3 (2)	6 (4)	44 (27)	59 (37)	49 (30)
In the last month, how often have you dealt successfully with day to day problems and annoyances?	29(18)	50 (31)	59 (37)	21 (13)	2 (1)
In the last month, how often have you felt that you were effectively coping with important changes that were occurring in your life?	24 (15)	47 (29)	54 (34)	29 (18)	7 (4)
In the last month, how often have you felt confident about your ability to handle your personal problems?	36 (22)	47 (29)	53 (33)	18(11)	7 (4)
In the last month, how often have you felt that things were going your way?	15 (9)	38 (24)	70 (43)	25 (16)	13 (8)
In the last month, how often have you found that you could not cope with all the things that you had to do?	10 (5)	15 (9)	69 (43)	43 (27)	24 (15)
In the last month, how often have you been able to control irritations in your life?	5 (3)	21 (13)	73 (45)	39 (24)	23 (14)
In the last month, how often have you felt that you were on top of things?	8 (5)	22 (14)	57 (35)	59 (37)	15 (9)
In the last month, how often have you been angered because of things that happened that were outside of your control?	9 (6)	16 (10)	48 (30)	47 (29)	41 (25)
In the last month, how often have you found yourself thinking about things that you have to accomplish?	0 (0)	8 (5)	23 (14)	38 (24)	92 (57)
In the last month, how often have you been able to control the way you spend your time?	33 (20)	36 (22)	49 (30)	33 (20)	10 (6)
In the last month, how often have you felt difficulties were piling up so high that you could not overcome them?	15 (9)	31 (19)	57 (35)	27 (17)	31 (19)

Figures in each cell depict number of responses and their percentage for each question in PSS

### Academic performance

The mean academic score of the students was 381 (SD = 42.26)). The mean academic score of the female students (n = 108) was 378.57 (SD 44.22); that of the male students (n = 53) was 387.15 (SD 37.71).

### Sources and self-rated severity of stressors

The most frequently [number of respondents (percentages)] occurring sources of stress reported by students as often/always were, 'high parental expectations' [101(63%)], 'frequency of examinations' [95(59%)], 'vastness of academic curriculum' [81(50%)], 'sleeping difficulties' [77 (48%)], 'performance in periodic examinations' [74(46%)], 'worrying about the future' [72(45%)], 'loneliness' [66(41%)], 'becoming a doctor (expectations on all fronts)' [64(40%)], 'Non availability of learning materials' [56(35%)], 'quality of food in mess' [56(35%)]. 'High parental expectations', 'frequency of examinations', 'sleeping difficulties', 'dissatisfaction with the class lectures', 'worrying about the future, 'vastness of academic curriculum/syllabus', and 'loneliness' were rated as most severe (Table 2).

**Table 2:** Response pattern of the 33 sources of stress and perceived severity (rated in a likert scale of 1-10) as reported by the students

	Frequency of occurrence			Severity	
Sources of Stress	Never/Rarely (%)	Sometimes (%)	Often/Always (%)	Median	IQR*
**Academic Stressors**					
Frequency of Examinations	20	21	59	6	3 - 8
Performance in Examinations	18	36	46	6	3 - 7
Academic Curriculum	20	30	50	5	3 - 7
Dissatisfaction with Class Lectures	29	42	29	5	3 - 7
Non-Availability of Adequate learning materials	39	26	35	5	2 - 7
Becoming a Doctor	30	30	40	5	2 - 8
Lack of time for recreation	36	29	35	5	2 - 7
Competition with Peers	43	23	34	4	1 - 7
Performance in practicals	65	16	19	2	1 - 4
Lack of special guidance from faculty	68	20	12	2	1 - 4
**Psychosocial Stressors**					
High Parental Expectations	24	13	63	7	3 - 10
Loneliness	30	29	41	5	2 - 8
Family Problems	46	29	25	4	1 - 7
Accommodation away from home	41	24	35	4	1 - 8
Political situation in the country	47	21	32	4	2 - 7
Relations with the Opposite Sex	55	21	24	3	1 - 7
Difficulty reading text books	63	26	11	3	1 - 5
Lack of entertainment in the institution and Lahore itself	52	24	24	3	1 - 6
Difficulty in the journey back home	49	17	34	3	1 - 7
Quality of food in mess	44	21	35	3	1 - 8
Financial strain	53	24	23	3	1 - 6
Inability to socialize with peers	63	20	17	3	1 - 5
Living conditions in the hostel	63	15	22	1	1 - 5
Member of fraternity or sorority	83	6	11	1	1 - 2
Lack of personal interest in medicine	76	14	10	1	1 - 3
Adjustment with roommate/s	69	10	21	1	1 - 5
**Health Related Stressors**					
Sleeping Difficulties	27	25	48	6	3 - 8
Class Attendance	49	23	28	4	1 - 6
Nutrition	43	25	32	4	2 - 7
Exercise	46	22	32	4	1 - 7
Quality of food in mess	44	21	35	3	1 - 8
Physical disability	63	21	16	2	1 - 5
Alcohol/Drug abuse/Smoking	86	2	12	1	1 - 1

*Inter Quartile Range

The correlation between perceived stress and academic performance was assessed using Pearson correlation coefficient; this was negative and not significant (r = -0.099, p > 0.05).

### Determinants of stressed-cases by logistic regression

By logistic regression analysis stressed cases were associated with being a female [OR 2.25, 95% CI 1.13-4.49], occurrence of psychosocial [OR 5.01 (95%CI 2.44-10.29)] and academic-related stressors [OR 3.17 (95%CI 1.52-6.68)] (Table 3).

**Table 3:** Determinants of stressed cases by logistic regression

Determinants	Number	Number of stressed Cases (%)	Univariate OR (95% CI)	Adjusted OR* (95% CI)
**Age (in completed years)**				
< 21	95	67(71.2)	1	1
>=21	66	41(62.1)	0.69 (0.33-1.4)	0.69 (0.35-1.33)
**Gender**				
Male	53	29(54.7)	1	1
Female	108	79(73.1)	**2.25 (1.07-4.76)**	**2.25 (1.13-4.49)**
**Batch of Study**				
First	83	55(66.3)	1	1
Second	78	53(67.9)	1.08 (0.53-2.20)	1.08 (0.56-2.08)
**Nationality**				
Pakistan	121	81(66.9)	1	1
International	40	27(67.5)	1.03 (0.45-2.36)	1.03 (0.48-2.19)
**Marital Status**				
Single	132	85(64.4)	1	
In a relationship	29	23(79.3)	2.12 (0.75-6.28)	2.12 (0.81-5.57)
**Medium of teaching in School**				
English	148	98(66.2)	1	
Urdu	13	10(76.9)	1.70 (0.41-8.19)	1.70 (0.45-6.46)
**HSSC**				
A-levels	40	30(75)	1	
Fsc and HSD	121	78(64.4)	0.21 (0.09-0.49)	0.62 (0.28-1.41)
**Day Scholar/Boarder**				
Day Scholar	99	68(68.7)	1	
Boarder	62	40(64.5)	0.83 (0.40-1.71)	0.83 (0.42-1.62)
**Occurrence of Psychosocial Stressors**				
Less than often	50	19(38.0)	1	
Often/Always	111	87(78.3)	**5.91 (2.69-13.13)**	**5.01 (2.44-10.29)**
**Occurrence of Academic Stressors**				
Less than often	97	54(55.7)	1	
Often/Always	64	52(81.3)	**3.45 (1.55-7.8)**	**3.17 (1.51-6.68)**
**Occurrence of Health Stressors**				
Less than often	147	97(65.9)	1	
Often/Always	14	9(64.2)	0.93 (0.26-3.39)	0.87 (0.28-2.75)

*Adjusted with Age, Gender, Batch of Study, Nationality, Marital Status, Medium of teaching in school, HSSC, Day Scholar/Boarder, Occurrence of Psychosocial Stressors, Occurrence of Academic Stressors, Occurrence of Health Stressors

## Discussion

In our study, we evaluated perceived stress among medical students including its sources and severity along with its correlation with academic performance, which may be of importance to both medical teachers and psychologists. To our knowledge such a detailed study has not been reported from Pakistani medical schools. In our study, medical students reported a higher level of perceived stress, which was significantly higher among female students, but it's correlation with self-reported academic performance was not significant. Academic and psychosocial stressors were reportedly more common both in terms of frequency of occurrence and severity. By logistic regression analyses stressed cases were associated with female gender, psychosocial and academic related stressors.

Life of a medical student or a health care professional can be very stressful. Mild, moderate, and high levels of stress and even burnout have been reported amongst medical students and health care professionals from other countries [25-29]. Previous studies have shown that medical school stress is a good predictor of nervous symptoms even when psychosocial variables such as marital status or cohabitation status, confident and other general self-esteem are taken into consideration [6]. Apart from all this, end-of-the-year first year medical students have shown to be worse off psychosocially than they were when they entered [30].

The amount and severity of stress experienced by medical students may vary according the settings of the medical school, the curricula, evaluation (examination) system etc. Previous studies from medical schools in different countries have reported varying levels of stress [1,5,6,9,13,15,16,18]. A study from Agha Khan University, Pakistan has reported that more than 90% of its students experienced stressed at one time or the other during their course [18]. A similar study from India reported that 73% of the students had perceived stress at some point or the other during their medical schooling [15]. Saipanish reported that 61.4% of students in a Thai Medical School had come across some degree of stress as calculated by the Thai Stress Test [16]. These studies have used different instruments to measure stress. This limits the comparability among these studies. We chose the perceived stress scale since this instrument has been documented for its reliability and validity [19,31-42]. The advantage of PSS is that it can be applied to a wide range of settings, to different subject types and includes items measuring reactions to stressful situations as well as measures of stress [19,31-42]. An important limitation of other reviewed stress scales for health professions students is that it focuses only on academic stressors, and lack of inclusion of personal issues or reactions to stressful situations (psychosocial issues), and poor applicability to broader settings.

In our study, sample proportion (67.08%) of the female students was higher than their male (32.92%) counterparts. Mean PSS scores among female students was significantly higher than that of male students. A plausible reason may be the conservative nature of society in Pakistan, where women lack freedom to participate in extra-curricular activities owing to the restrictions imposed on them by the society. However, Cohen has reported that there was no significant difference in stress using PSS between male and female students. In addition, private life may conflict with professional life and this may cause stress. We lacked sufficient information, which could assist us in carrying out further analysis about this. The overall average stress score in our sample was significantly higher (p < 0.05) than that reported by Cohen [31].

In our study the correlation between perceived stress and academic performance was negative and not statistically significant. This finding is contrary to existing literature which states that acute stress is a predictor of reduced scholastic performance, especially in examinations [9,43]. Plausible explanations for such contradictory reports could be that only acute stress but not longstanding stress may affect academic grades during examinations. In addition, the students who are striving to perform well in examinations may make themselves stressed. Individual coping styles and skills along with their access to different forms of social support may have played a role in negating the effect of stress on academic performance; this could not be explored in our study.

Most students had experienced either academic and/or psychosocial stressors. Among academic stressors, 'tests/exams' were the chief sources of stress. Despite this, test/exams are important in the medical training as a standard for evaluation/assessment. Also examinations encourage students' learning and also provide feedback to the teachers. Those students who perceive tests/exams as a burden may experience stressful situations while for others, who consider exams useful, they may assist in their learning. Previous studies have also reported that academics/exams are common sources of stress among medical students [9,15-19,44-47]. There may be a need to revisit the evaluation/examination system to make it less stressful to the students. An important source of stress was related to psychosocial factors. This may be due to time constraints for self, family, friends and entertainment due to the demanding medical curriculum. Another reason could be inadequate recreational facilities provided by the college as reported by the students. The students, who were stressed, reported that psychosocial and academic related stressor groups had occurred more frequently. This was revealed by the association of these factors with stressed cases by logistic regression analysis. This may suggest that students had a global response to a wide range of potential stressors rather than being limited to a few specific items. However, there was a high odds ratio on both univariate and multivariate analysis suggesting high collinearity. This suggests that students having academic stressors were more likely to have psychosocial stressors as well or vice versa. A study from USA has recommended that teaching stress management and self-care skills to medical students may prove to be beneficial [48]. There is a need to look at the applicability of such measures which are feasible in our medical school setting.

## Limitations

Lack of generalisation of our results to other medical schools in Pakistan is an important limitation of this study. Since the information was collected on self-administered questionnaires/instruments we cannot rule out information bias. The use of self-reported academic scores is another limitation and future studies should use their academic records. Cross-sectional design of our study is yet another limitation since associations presented lack temporality. Prospective studies are necessary to study the associations between occurrence stressors and incidence of stress. Though response rates (80%) were fairly good there could be some selection (non-response) bias. Though participation was anonymous, authors had the opportunity to meet the non-respondents and ask them reasons for not participating in the survey. The main reasons that surfaced were the sensitive and personal nature of the study as well as the length of questionnaire. The other reasons were absence from college due to sickness or travel on the day of the survey.

## Conclusion

The students reported higher levels of stress. The most frequently occurring stressors among the students were related to academic and psychosocial domains. The associations between stressed cases and female gender, occurrence of academic and psychosocial stressors needs to be further tested by prospective studies.

## Competing interests

The authors declare that they have no competing interests.

## Authors' contributions

MS conceived, designed and carried out the study and prepared the first draft of the manuscript. SH helped design and carrying out the study. SM helped in carrying out the study, performed data analysis, and helped in drafting the manuscript. CTS assisted in design, interpretation of results, drafting the manuscript and critically evaluated earlier drafts of the manuscript. All authors read and approved the final manuscript.

## Pre-publication history

The pre-publication history for this paper can be accessed here:

http://www.biomedcentral.com/1472-6920/10/2/prepub
